# Visual search and childhood vision impairment: A GAMLSS-oriented multiverse analysis approach

**DOI:** 10.3758/s13414-023-02670-z

**Published:** 2023-02-23

**Authors:** Paul A. Constable, Lynne Loh, Mallika Prem-Senthil, Fernando Marmolejo-Ramos

**Affiliations:** 1 College of Nursing and Health Sciences, Caring Futures Institute, Flinders University, Bedford Park, South Australia Australia; 2 Centre for Change and Complexity in Learning, The University of South Australia, Adelaide, South Australia Australia

**Keywords:** Visual search, Reaction time methods, Statistical inference

## Abstract

The aim of this report was to analyze reaction times and accuracy in children with a vision impairment performing a feature-based visual search task using a multiverse statistical approach. The search task consisted of set sizes 4, 16, and 24, consisting of distractors (circle) and a target (ellipse) that were presented randomly to school-aged individuals with or without a vision impairment. Interactions and main effects of key variables relating to reaction times and accuracy were analyzed via a novel statistical method blending GAMLSS (generalized additive models for location, scale, and shape) and distributional regression trees. Reaction times for the target-present and target-absent conditions were significantly slower in the vision impairment group with increasing set sizes (*p* < .001). Female participants were significantly slower than were males for set sizes 16 and 24 in the target-absent condition (*p* < .001), with male participants being significantly slower than females in the target-present condition (*p* < .001). Accuracy was only significantly worse (*p* = .03) for participants less than 14 years of age for the target-absent condition with set sizes 16 and 24. There was a positive association between binocular visual acuity and search time (*p* < .001). The application of GAMLSS with distributional regression trees to the analysis of visual search data may provide further insights into underlying factors affecting search performance in case-control studies where psychological or physical differences may influence visual search outcomes.

The aim of this brief report was to investigate the impact of a vision impairment on search efficiency using a classical feature-based search paradigm (Green, 1991) with an alternative statistical analysis using distributional regression trees (Schlosser et al., 2019). Common causes of vision impairment in children include inherited retinal dystrophies, congenital glaucoma, retinopathy of prematurity, and albinism, which are frequently associated with nystagmus (Rahi, 2007; Teoh et al., 2021), and may impact the child’s ability to read at a similar speed to their typically sighted peers (Loh et al., 2021). Studies in amblyopia have demonstrated worse performance for feature and conjunctive search strategies (Tsirlin et al., 2018) that are directly related to oculomotor control (Chen et al., 2018; Huurneman et al., 2014) and crowding (Levi, 2008).

Visual search studies may involve complex populations where multiple underlying factors such as visual acuity, oculomotor control, or cognitive ability may impact their search ability and contribute to the overall results. For example, in studies involving neurodevelopmental disorders such as autism spectrum disorder (ASD) where attention (Scheerer et al., 2021), oculomotor control (Pruett Jr et al., 2013), sex (Harrop et al., 2019), crowding (Lindor et al., 2018), diagnostic procedure (Almeida et al., 2010), age of the population (Constable et al., 2010), or search strategy interpretation (Keehn & Joseph, 2016) may all impact on overall performance and interpretation of the results. Similarly, in studies involving patient groups with an acquired loss of function due to neurodegeneration such as dementia or vision impairment through disease such as glaucoma or age-related macular degeneration, similar group characteristics such as the duration of the vision impairment, cortical evoked potentials, contrast, cognitive ability, or extent of visual field may be additional factors that impact on search performance (Lee et al., 2020; Sklar et al., 2020; Vullings et al., 2022; Xue et al., 2022) but are not readily analyzed using standard statistical analyses. Thus, the application of additional statistical analyses to search data may help in identifying underlying factors that contribute to the overall search performance in complex populations involved in case-control studies.

Whilst it is intuitive to presume that search performance will be affected in individuals with a vision impairment, there have been few studies that have explored visual search in a school-age population of individuals with a vision impairment. To confound the findings, different search tasks have been adopted to explore the impact of vision impairment on search efficiency. In a real-world paradigm, children with a vision impairment were slower to locate an object within a natural scene (i.e., a stapler in an office space; Tadin et al., 2012). Longer search times have also been demonstrated in children and adults with a vision impairment in feature-based and conjunctive search tasks that increases with the number and complexity of the distractors which is associated with the degree of oculomotor control, crowding, and extent of their functional visual field (Huurneman et al., 2014; Kuyk et al., 2005).

Further underlying factors that may affect search speed and accuracy in typical observers are attention that guides visual search based on salient features, prior experience, and the scene context (i.e., where an object of interest is most likely to be found; Wolfe, 2021). The functional field of vison has become a theoretical framework in which to describe visual search with the extent of the functional field of vision determining the number of fixations required to determine whether a feature is present or absent (Hulleman & Olivers, 2017). Distortions in the peripheral functional field of vision may enhance confusions between a target and distracter and reduce search performance (Zhang et al., 2015). These factors may play an important role in search efficiency in children with a vision impairment where visual field size and oculomotor control may be compromised and exhibit slower reading speeds (Webber, 2018). For a recent review of visual search theories, see Wolfe (2020).

Typically, a feature based visual search study would compare the reaction times, accuracy, and the slope and intercept of the reaction time versus set-size relationship to draw conclusions about search strategy and performance between groups. The slope of the relationship is related to search efficiency with the intercept to attention (Sternberg, 1966). In this study, a different approach was adopted to the analysis of visual search reaction times and accuracy for children with and without a vision impairment. Reaction times and accuracy were analyzed using a multiverse approach in which key variables were identified via algorithms for variable selection and subsequently used in a distributional regression tree (Schlosser et al., 2019); this method combines GAMLSS (generalized additive models for location, scale, and shape) modelling (Stasinopoulos et al., 2018) with decision trees. In brief, decision trees are tools in the form of directed graphs designed to support decisions based on ‘if-then’ conditional statements (e.g., if Condition 1, then outcome; see Breiman et al., 1984; Kamiński et al., 2017; Quinlan, 1987). GAMLSS, on the other hand, is a semiparametric regression framework capable of dealing with nonlinear relationships via smoothers and adopting any probability distribution to represent the response variable. That is, GAMLSS allows performing ordinary linear, generalized linear, and generalized additive regression but with the possibility of inspecting the effects of covariates on all the parameters of the statistical distribution representing the dependent variable (Marmolejo-Ramos et al., 2022).

In visual science, GAMLSS has been applied to generating reference curves for refractive errors (Truckenbrod et al., 2020) and to identify outliers with a population of children whose developmental profile of visual acuity differs (Stander et al., 2019). By applying a different analytical approach to this standard search task, the study aimed to identify variables that may be important to visual search in children with and without a vision impairment. These findings may be applicable to other studies in which multiple factors may affect search efficiency owing to physical or psychological differences between study populations. Such variables may include, but not limited to, attention (Scheerer et al., 2021), saccade time (Vullings et al., 2022), age (Borges et al., 2020; Xue et al., 2022), sex (Harrop et al., 2019), visual field size (Wiecek et al., 2012), visual acuity (Kuyk et al., 2005), oculomotor control (Chen et al., 2018; Huurneman et al., 2014), clinical diagnosis severity in the case of neurological disorder (Almeida et al., 2010), or crowding (Levi, 2008). Thus, GAMLSS-based decision trees may provide a useful method to identify factors that may influence search performance depending on the task.

## Methods

### Participants

The inclusion criteria for the vision impairment group were individuals 5 to 18 years of age with a diagnosis of vision impairment by an ophthalmologist and registered as either blind or visually impaired. Exclusion criteria were participants with co-occurrence of a neurodevelopmental disorder such as autism spectrum disorder, dyslexia, or intellectual disability and were unable to follow simple verbal instructions. Corrected binocular acuity was limited to the range of 6/9.5 to 6/190 (LogMAR 0.2 to 1.5).

A total of 39 vision-impaired individuals were recruited from the South Australian School for Vision Impaired, of which the majority of 32 (82%) had nystagmus. The mean ± *SEM* binocular visual acuity of the vision impaired group was 0.63 ± 0.05 LogMAR with range of 0.21 to 1.46 (~6/9.5–6/180) measured using the Freiburg acuity test using four alternative forced choice strategy with Landolt rings (Bach, 1996). Comparison control children (*n* = 33) were recruited from local schools and friends and family who had normal to near normal corrected acuities and no oculomotor imbalances. There were no significant differences between groups for age (mean ± *SEM*), vision impaired = 11.6 ± 0.5 and control = 13.0 ± 0.5 years; Mann–Whitney *U*, *p* = .052 and sex/gender (vision impaired male *n* = 22 [56%] and control *n* = 19 [61%]); χ^2^(1) = .03, *p* = .87. The vision-impaired group consisted of individuals with a diagnosis of albinism (*n* = 12), inherited retinal disease (*n* = 12), visual pathway disorder (optic nerve/cortical; *n* = 6), congenital nystagmus (*n* = 3), congenital cataract (*n* = )2, and *n* = 1 each of retinopathy of prematurity, myopia, anterior uveitis/cataract, or vision impairment with unknown etiology. This study was performed in line with the principles of the Declaration of Helsinki and was approved by the Women’s and Children’s Health Network, Human Research and Ethics Committee (HREC/19/WCHN/177), and The South Australian Department of Education (2019-0047). Written informed consent was obtained from the parent/guardian of the participant prior to taking part in this study.

### Visual search

**Fig. 1:**
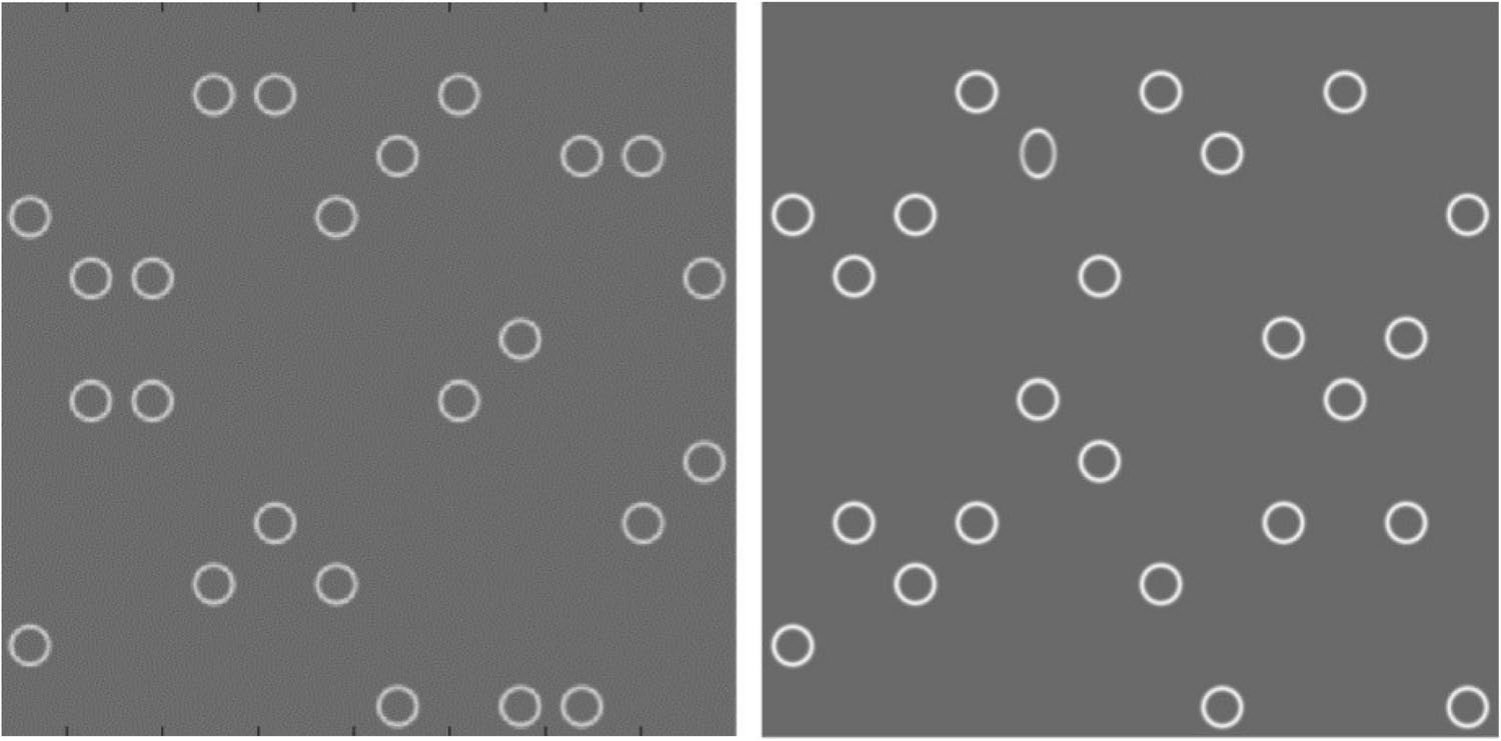
Display presentations for the feature search task with set size 24. Left figure shows the target-absent condition (no ellipse), and the right figure shows the target-present (ellipse) condition, in which an ellipse is present within the circular distractors

Methods for this study have been previously described in Constable et al. (2010). The target (ellipse) and distractor (circle) were presented on the display of a MacBook Air-11.6-inch display (1,366 × 768 pixels at 75Hz) with mean luminance of 65 cd.m^-2^ and the subject sitting comfortably at a viewing distance of 40 cm in dim room illumination (150–200 lx). Experiments were run using MATLAB (The MathWorks, Natick, MA, USA) and Psychophysics Toolbox extensions for stimulus generation, experiment control and recording the participant’s responses (Brainard, 1997; Pelli, 1997). The circle distractors had dimensions of 0.8 cm × 0.8 cm, projecting a visual angle of 1.15° whilst the target ellipse had dimensions of 0.7 cm × 1.0 cm, projecting a visual angle of 1.00° and 1.43° (Fig. 1). The stimuli were further refined via anti-aliasing process through a Gaussian blur kernel (*σ* of 2 pixels) which reduced image noise and smoothed the border of the circles and ellipse.

The stimuli were equally spread on a 12 × 12 grid to allow equal number of stimuli on each side from the point of fixation. Three set sizes were used with either target present or absent consisting of 4, 16, and 24 items displayed randomly within the grid and presented randomly for each trial. At the start of each trial the participant was instructed to press the key ‘P’ if the ellipse was present or the key ‘A’ if the ellipse was absent. A total of 80 presentations were performed in each trail (8 practice plus 72 test trials). The participant was instructed to do the task as quickly and as accurately as possible.

### Analysis

In this study, eight practice and 72 test trials were administered in random order for each participant. The first eight presentations were used as practice trials and were excluded from the analysis. During the practice trials, the instructor (LL) gave verbal feedback and an audible cue sounded for an incorrect response during the practice or test trials so that the participant had feedback throughout the recording session. For the test trials there were six possible combinations of set size (4, 16, or 24) and target (present or absent) which allowed for 12 responses on average (range 8–15) per possible combination of set size and target (72 trials/6 possible combinations =12). Given the random number of total presentations of each test condition which ranged from 8 to 15, we included only the first eight correct reaction times for each combination of the test trails. Test trials were excluded if the reaction time was greater than 10 seconds. The accuracy (percentage correct) was equal to 8 divided by the number of trial combinations required to obtain the eight correct responses. (An accuracy of 83% would equate to 10 correct from 12 trials.) If the accuracy was less than or equal to 50% (i.e., less than or equal to chance) then this observation was excluded. In total six observations were excluded from the vision impaired group for failing to obtain an accuracy of greater than 50%.

### Statistical modelling

Reaction time and accuracy data were analyzed separately, and the variables considered were group (G; c = control, I = impaired), gender (g; m = male, f = female), set size (S; 4, 16, and 24), target (T; ab = absent, p = present), nystagmus (n), and age (a). The full model analyzed for each variable was DV ~ G × S × T + n + a + g; such that ‘DV’ stands for either reaction times or accuracy data, ‘*’ stands for interactions and main effects and ‘+’ stands for main effects only.

The statistical modelling consisted of (1) determining the importance of the variables via the Boruta (Kursa & Rudnicki, 2010) and the One Rule (Holte, 1993) algorithms; (2) examining the full model via quantile (Waldmann, 2018), robust (Yu & Yao, 2017), and distributional (Kneib et al., 2021) regression models (this step is grounded in a multiverse analytical approach, as described by Steegen et al., 2016, akin to ensemble methods in machine learning, and allows rectifying Step 1 and finding patterns in data); (3) proposing an explainable reduced model based on the patterns found in Step 2 and examining that model through the regression techniques used in Step 2 (additionally, if suitable, a factorial design version of the explainable model was analyzed via ANOVA-type statistic (Brunner et al., 2017; otherwise the model was analyzed via quantile regression); and (4) generating a distributional regression tree as described by Schlosser et al. (2019).

Different from traditional regression trees, distributional regression trees enable to fit continuous and discrete variables’ location, scale, and shape parameters with probability distributions other than those in the exponential family (which includes the Normal distribution). Each distribution’s parameter is fitted with the same covariates and the first split occurs based on the parameter that shows the largest effect (no smoother can be applied to numeric covariates; i.e., the tree is based on proper distributional differences). Distributional trees detect split points which correspond to detecting abrupt changes precisely, but this might be a problem when the underlying effect is smooth. If this is the case (i.e., if the underlying effect is smooth), it is usually a good idea to build not only a single distributional regression tree but an ensemble of trees, i.e., a distributional forest. Additionally, the results of regression trees are interpretable and enable predictions and decisions relating to the data at hand (see Chapter 8 in James et al., 2021, and Section 5.4 in Molnar, 2022). Thus, the goal of the statistical modelling in this study is to propose a regression tree that preserves a small set of key variables and that ultimately facilitates a parsimonious interpretation.

As the name indicates, distributional regression trees are dependent on GAMLSS regression. GAMLSS is one of the few existing distributional approaches able to overcome focusing on average or mean differences (Kneib, 2013; Kneib et al., 2021). More specifically, GAMLSS is a semiparametric approach for statistical learning and modelling that allows dealing with random effects and nonlinear covariates via additive terms (e.g., smoothers; this is the ‘semi-’, or nonparametric, part of GAMLSS) and fitting the dependent variable with any probability distribution (this is the ‘parametric’ part of GAMLSS). While using smoothers for numeric covariates is characteristic of generalized additive models (Hastie & Tibshirani, 1986), GAMLSS offers that option in addition to enabling to inspect the effect of covariates on all the parameters of the dependent variable. Thus, while a traditional linear regression assumes the dependent variable follows a Normal distribution and allows to determine mean differences or effects, GAMLSS would allow determining mean effects and effects on the data’s variability. This is so, given that the Normal distribution has the parameters mu and sigma that represent the data’s location and scale (in the case of the Normal distribution there is only one shape as its skewness and excess kurtosis are always 0). In other words, provided the Normal distribution is a good fit to the response variable, only GAMLSS allows to examine if there are effects of the covariates on the data’s location and scale.

Appropriate nonparametric tests were used as required to compare demographic data. Association between reaction time and visual acuity were evaluated using Kendall tau statistic and the percentage bend correlation estimator (Wilcox, 1994). A *p* value of <.05 was taken as significant.

All data, R code, statistical outputs, and MATLAB stimulus code are available (https://figshare.com/projects/Feature_visual_search_in_children_with_a_visual_impairment_A_multiverse_analysis_approach/132971).

## Results

### Reaction times

The final set of variables arrived at from Steps 1–3 consisted of G, S, T, and g. The ANOVA-type statistic (ATS) was used given that the factorial design version of the explainable model enabled the variable ‘gender’ to interact with the other variables and because factorial designs are interpretable. The ATS suggested main effects of all factors, G = *F*(1, 305) = 210.2, *p* < .0001, S = *F*(1.99, 305) = 136.9, *p* < .0001, T = *F*(1, 305) = 128.6, *p* < .0001, and g = *F*(1, 305) = 18.2, *p* < .0001, and two significant two-way interactions, T × S = *F*(1.99, 305) = 8.51, *p* < .001, and G × g = *F*(1, 305) = 6.71, *p* = .01. All other interactions and main effects were nonsignificant (*p* > .11).

**Fig. 2:**
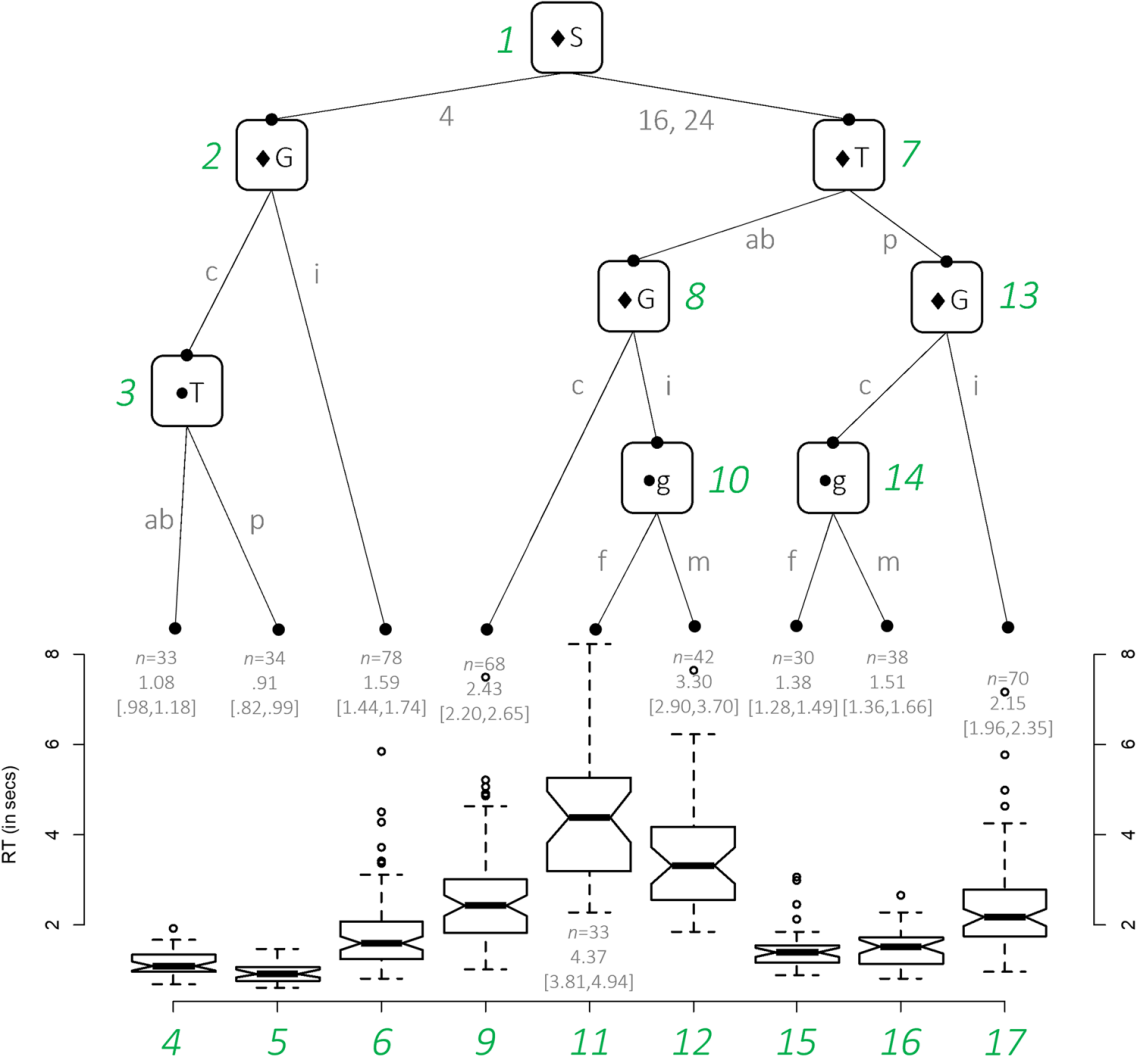
Distributional regression tree for reaction times (RTs) according to the factors group (G; c = control, i = impaired), gender (g; m = male, f = female), set size (S; 4, 16, and 24), and target (T; ab = absent, p = present). The data were modelled with an ExGaussian distribution. ● *p* < .05 and ♦ *p* < .001. The numbers in green show the order of the nodes. The number of observations, the median and approximate 95% CI around the median are reported, respectively, in the format *n* = xx, xx [xx, xx]. (Colour figure online)

Figure 2 shows the distributional regression tree, with the upper ‘top’ branches having greater significance than the lower ‘bottom’ branches based on the differences in their location, scale, and shape. Thus, the first node is the starting point for decisions is between the distributions of Set Size 4 and Set Size 16, 24. For the case of Set Size 4, the distributions differ between group (Node 2) but only in the case of controls is there a significant difference between the distributions of reaction times in the target-absent and target-present condition (Node 3). The distributions of the (conditional) reaction time data are shown as Nodes 4–6.

There was a significant (*p* < .001) positive correlation between reaction time and binocular visual acuity (Kendall’s tau = .34 and percentage bend correlation = .44).

### Accuracy

**Fig. 3:**
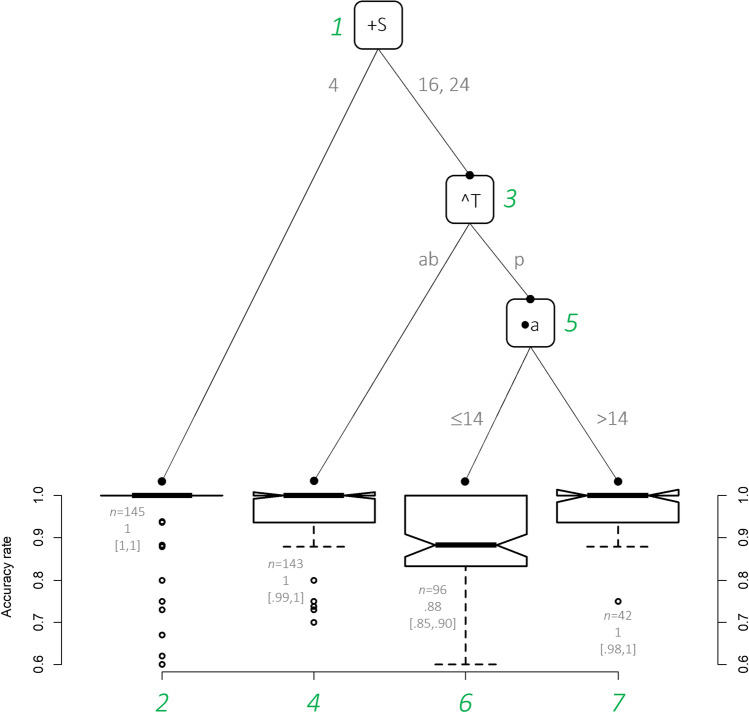
Distributional regression tree for accuracy rates according to the factors age, set size (S; 4, 16, and 24), and target (T; ab = absent, p = present). The data were modelled with a generalized beta Type 1 distribution. ●*p* < .05, **^***p* = .005, and **+***p* = .002. The numbers in green show the order of the nodes. The number of observations, the median and approximate 95% CI around the median are reported, respectively, in the format *n* = xx, xx [xx, xx]. (Colour figure online)

The final set of variables arrived at from Steps 1 to 3 consisted of S, T, and a. An ANOVA-type statistic was not suitable to this data given that ‘age’ is not a categorical variable and rendering it into a categorical one, although possible, is not recommended (see Gelman & Park, 2009). A linear quantile model was thus used. This model suggested one two-way (T × S = *β*: −.10, *p* = .02) and one three-way significant interactions (T × S × a = *β*: 6.3*e*^-03^, *p* = .03) only. Figure 3 represents the way these variables interact.

The distributional regression tree indicates that the most significant difference (Node 1) was between accuracy scores between Set Size 4 and Set Sizes 16, 24 (*p* = .002). Within Set Sizes 16, 24 the next most significant difference between the distributions was for the target-present or target-absent condition (*p* = .005). The next most significant difference between the distributions that influenced accuracy was age, with the participants aged under 14 being significantly less accurate (*p* = .03). There were no significant overall differences between groups for accuracy in either condition in a linear quantile model that considered main effects of and interactions among G, S, and T while controlling for ‘age’ (*p* = .73).

## Discussion

This study adopted a different statistical approach, based on comparisons of the location, shape, and scale of data’s distributions to analyze the most significant differences between reaction times and accuracy in school aged individuals with a vision impairment and typically sighted children. For reaction times, based on Fig. 2, the main finding was that there were significant differences in search performance based on either Set Size 4 or on Set Sizes 16 and 24 (*p* < .001) at Node 1. This observation implies that the group’s performance depended upon set size above other factors. Following to Node 2, there is a group difference for reaction times at this set size (*p* < .001) with the vision impairment group having a median reaction time of 1.59 ms for target absent or present. However, for the control group there was a significant difference for reaction time (*p* < .05) between target present (0.91 ms) or absent (1.08 ms) at Set Size 4 (Node 3), which was not significant for the vision impairment group. These differences at Set Size 4 suggest that the search time for target absent or present are not different for the vision impairment group implying a serial search strategy for target-present and target-absent conditions with equivalent search times (Nodes 4–6). In contrast, the control group had a significantly shorter search time as expected for the target-present condition as they would halt the search once the target was located.

For Set Sizes 16 and 24, the observations were different in some respects. Whilst there was a significant difference based on target present or absent (*p* < .001) at Node 7, this is consistent with standard findings in feature search based on serial and parallel search models (Duncan & Humphreys, 1989; Treisman & Gelade, 1980). For the target-absent condition at Set Sizes 16 and 24 (Node 8), the control group were significantly faster (*p* < .001) than the vision-impairment group, with a median search time of 2.43 seconds (Node 9). The unexpected observation was that for sex (Node 10) the female vision impaired group were significantly (*p* < .05) slower 4.37 seconds (Node 11) than males 3.10 seconds (Node 12). These findings suggest that vision-impaired females take longer in their approach to a target-absent search strategy, and perhaps taking longer to confirm that the target ellipse is absent from the circular distractors before committing to a response.

For the target-present condition at Set Sizes 16 and 24, there were significant group differences, with the vision-impairment group performing slower overall (2.15 seconds Node 17) than the control group (*p* < .001; Node 13). However, in contrast to the target-absent condition, where vision-impaired females were slower, in the target-present condition, it was the control males (Node 14) that were significantly (*p* < .05) slower (1.51 seconds) than their female (1.38 seconds) counterparts (Nodes 15 and 16). This observation is unlikely to be due to differences in hemispheric volume, with males performing poorly in a feature-based task when the target is in the left hemifield compared with the right (English et al., 2021) because target and distractors were of equal number in each hemifield for this task. This observation may be that in this cohort the male subjects took longer to decide on whether the ellipse was present or not.

The exact reason as to why there were sex differences in reaction time depending on Set Sizes 16 and 24, for the control (target-present males slower [Node 14]) or vision impairment (target-absent females slower [Node 10]) is not clear, given the target distractor arrays were symmetrical about the midline. The observation may indicate some differences in the cognitive style of the participants based on their ability and confidence to decide whether a target was present or absent. We did not adjust for the individual’s discrimination thresholds to detect a circle from the ellipse or estimate their critical spacing and the possible effects of crowding on search performance which was a limitation of the study Constable et al. (2010). These factors may have also contributed to sex differences observed. Nonetheless, the application of the distributional regression tree approach to reaction times provides a hierarchical view of the main factors that differentiate the groups based on set size (Node 1) as the most important to differentiate the reaction times. As expected, there was a significant (*p* < .001) positive correlation (percentage bend correlation = 0.44) between reaction time and visual acuity, with reduced visual acuity resulting in longer search times in adults and children as previously reported (Huurneman et al., 2014; Kuyk et al., 2005; Luo et al., 2012).

With respect to accuracy (Fig. 3), we found that there was a significant difference based on set sizes (4 compared with 16 and 24) at Node 1 (*p* = .002). This finding is as expected, with search accuracy decreasing with the set size and number of distractors. However, there were no significant differences between groups in any of the nodes for accuracy indicating that the control and vision impairment groups performed with equal accuracy across combinations of set size and target absent or present. Only at Node 3 for Set Sizes 16 and 24, there was a significant difference between the overall accuracy for the target-present compared with the target-absent condition (*p* = .005) which is consistent with feature search findings with greater errors when the target is present than when it is absent (Duncan & Humphreys, 1989; Treisman & Gelade, 1980).

The interesting observation occurred at Node 5 in the target-present condition for Set Sizes 16 and 24, where participants less than 14 years of age were significantly less accurate (88%) (*p* < .05) than those over 14 years of age (98%). These age differences may be related to differences in the rate of maturation of visual search networks that have been identified using imaging studies in children ages 7 to 16 (Lidzba et al., 2013). Gil-Gómez de Liaño et al. (2020) found a similar result for accuracy in typically developing children with accuracy reaching a plateau level at approximately age 9–10 for a complex search task using real-life objects. Thus, for accuracy analysis, the hierarchical structure of the regression tree gives an overview of the most significant factors affecting accuracy, which was set size followed by target present or absent, then age. With respect to these populations, the inference is that a vision impairment does not significantly impact search accuracy compared with school age matched individuals.

The methodological approach proposed herein is based on the GAMLSS framework. GAMLSS has been adopted for growth charts (e.g., Borghi et al., 2006), brain charts (e.g., Bethlehem et al., 2022), and reference curves (see the Introduction; see also Durán et al., 2016). More recently, GAMLSS has been featured to model psychological (Campitelli et al., 2017) and educational (Wiedermann et al., 2022) data. As mentioned above, GAMLSS overcomes limitations of other techniques, such as ordinary linear regression and generalized linear regression, by taking care of nonlinear covariates and relating the conditional mean of the response to explanatory variables through distributions other than those of the Exponential family. GAMLSS is also an improvement on generalized additive models by allowing to model all the parameters of the response variable. The novel attribute that only GAMLSS can offer is to assess the effects of covariates on all the parameters of the statistical distribution that best fits the response variable.

While existing decision trees can only produce one flowchart-like structure for a specific model, GAMLSS-based decision trees can have different structures. This is so because the flowchart-like structure of the tree will change depending on the distribution used to model the dependent variable in the GAMLSS model. In the present study, the reaction times were modelled via the Ex-Gaussian distribution as this is the most common distribution used to fit such type of data (Marmolejo-Ramos et al., 2015). However, reaction time data from a potential replication study could be better fitted with other candidate distributions such as, just to mention a few, the Gamma, Ex-Wald, or Birnbaum–Saunders. In those cases, the decision trees would be different, but they will likely highlight common patterns in the data.

In conclusion, GAMLSS may provide additional information to studies using visual search in addition to standard measures of set-size slopes and intercepts that are traditionally used in visual search tasks to evaluate serial or parallel search strategies (Kristjánsson, 2015; Wolfe, 2016). For example, in this simple case, the factors of age and gender were important in identifying search performance differences between children with and without a vison impairment at specific set sizes and in cases of target present or absent. Similarly in search studies involving participants where neurodevelopmental or neurodegenerative conditions may impact search ability and performance such as autism spectrum disorder (Almeida et al., 2010; Constable et al., 2010, 2020; Gregory & Plaisted-Grant, 2016; O’Riordan, 2004), attention-deficit/hyperactivity disorder (Seernani et al., 2021), dementia (Douglass et al., 2019), and Parkinson’s disease (Ranchet et al., 2020). In such case-control studies, the application of GAMLSS with distributional regression trees to determine the most to least significant factors affecting reaction time and accuracy may yield new insights into the differences between case and control groups.

## Data Availability

The datasets analyzed during the current study are available in the FigShare repository: https://figshare.com/projects/Feature_visual_search_in_children_with_a_visual_impairment_A_multiverse_analysis_approach/132971
